# Phylogenetic identification of lateral genetic transfer events

**DOI:** 10.1186/1471-2148-6-15

**Published:** 2006-02-11

**Authors:** Robert G Beiko, Nicholas Hamilton

**Affiliations:** 1 Institute for Molecular Bioscience, The University of Queensland, Brisbane, Australia and ARC Centre in Bioinformatics, Australia; 2 Advanced Computational Modelling Centre, The University of Queensland, Brisbane, Australia

## Abstract

**Background:**

Lateral genetic transfer can lead to disagreements among phylogenetic trees comprising sequences from the same set of taxa. Where topological discordance is thought to have arisen through genetic transfer events, tree comparisons can be used to identify the lineages that may have shared genetic information. An 'edit path' of one or more transfer events can be represented with a series of subtree prune and regraft (SPR) operations, but finding the optimal such set of operations is NP-hard for comparisons between rooted trees, and may be so for unrooted trees as well.

**Results:**

Efficient Evaluation of Edit Paths (EEEP) is a new tree comparison algorithm that uses evolutionarily reasonable constraints to identify and eliminate many unproductive search avenues, reducing the time required to solve many edit path problems. The performance of EEEP compares favourably to that of other algorithms when applied to strictly bifurcating trees with specified numbers of SPR operations. We also used EEEP to recover edit paths from over 19 000 unrooted, incompletely resolved protein trees containing up to 144 taxa as part of a large phylogenomic study. While inferred protein trees were far more similar to a reference supertree than random trees were to each other, the phylogenetic distance spanned by random versus inferred transfer events was similar, suggesting that real transfer events occur most frequently between closely related organisms, but can span large phylogenetic distances as well. While most of the protein trees examined here were very similar to the reference supertree, requiring zero or one edit operations for reconciliation, some trees implied up to 40 transfer events within a single orthologous set of proteins.

**Conclusion:**

Since sequence trees typically have no implied root and may contain unresolved or multifurcating nodes, the strategy implemented in EEEP is the most appropriate for phylogenomic analyses. The high degree of consistency among inferred protein trees shows that vertical inheritance is the dominant pattern of evolution, at least for the set of organisms considered here. However, the edit paths inferred using EEEP suggest an important role for genetic transfer in the evolution of microbial genomes as well.

## Background

An unexpected observation from the early genome sequencing era has been the extent to which different sets of putatively orthologous genes often yield strongly supported but incompatible tree topologies. Whether this disagreement is due primarily to violations of phylogenetic assumptions or to lateral genetic transfer (LGT) is still the subject of fierce debate [1-6]. Where one or more LGT events are suspected as a cause of topological differences between trees, it is often desirable to identify the lineages implicated, and if possible the direction of transfer (precisely identifying the *donor *and *recipient *taxa) as well. The correct identification of these events depends on the size and shape of the trees being compared, and the position of the LGT events within these trees. When taken in aggregate, the LGT events implied by many trees can reveal information about major pathways of gene sharing between closely and distantly related organisms [7-9].

Deduction of LGT events from phylogenetic trees is often based on the implicit or explicit idea of a *reference *tree that accurately describes the evolutionary relationships between the organisms under consideration [9-11]. In the absence of statistical bias, complications of paralogy, and LGT, all strongly supported gene or protein trees would agree with this reference topology. Even if the reference tree is simply an amalgam of many trees based on molecular sequence (e.g., a *supertree*), the implied 'organismal' history will be represented as a strictly bifurcating hierarchy with a defined point of origin in time (the last common ancestor or root). *Test *trees inferred from a single gene or protein, or even a concatenated set of such sequences, will have no obvious rooting unless uniform rates of evolution (a molecular clock) are assumed, in which case the tree can be midpoint rooted [12], or if the inferred gene tree is sufficiently similar to the reference tree such that the root of the latter can be confidently assigned to the former. Since a strict molecular clock assumption is frequently violated by empirical data [13,14], and the rooting of test trees may not be obvious in the face of LGT and biased evolution, comparisons between a rooted reference tree and one or more unrooted 'test' trees have the strongest evolutionary justification and are more general than comparisons between two rooted trees.

Another important component of tree comparisons is the ability to deal with multifurcating nodes in trees that are incompletely resolved. While most traditional phylogenetic methods produce completely resolved, strictly bifurcating trees as output, the statistical support (often, bootstrap proportion or Bayesian posterior probability) at some of these bifurcating nodes may be extremely weak. Thus, a disagreement between two trees that is based on a weakly supported topological feature may be of no interest, and it is often preferable to collapse the corresponding feature into a multifurcating node. Finally, in broad phylogenomic studies most sets of putatively orthologous genes or proteins will not cover the entire reference tree, so it may be necessary to 'project' the reference tree by removing the non-represented taxa before performing the comparison. However, if inferred LGT events are to be compared, it must be possible to evaluate them all in light of the complete reference tree.

**Figure 1:**
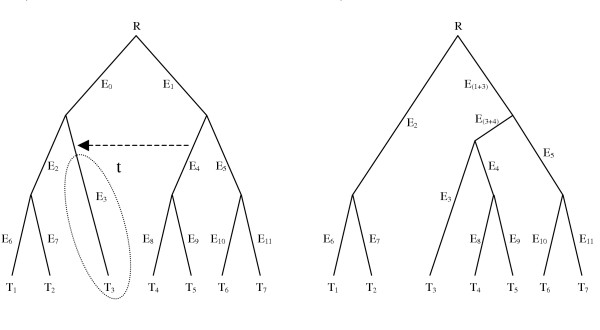
**SPR operation on a rooted phylogenetic tree**. The tree in panel (a) is subjected to an SPR operation, with participants and direction indicated with the dashed arrow. Edge E_4 _is the donor edge, which is split by acquisition of the recipient edge E_3_. Since they are no longer split by E_3_, edges E_0 _and E_2 _are consolidated into a single edge, which implies the same split of taxa as E_2_. Splitting E_4 _yields a new parent edge E_(3+4)_, and a child edge that implies the same split as E_4_. Finally, the bipartitions implied by every other edge up to the common ancestor of the donor and recipient edges (in this case, the root R) are modified by the swapping of subtree *t *from one partition to the other. Thus, in this case E_1 _now implies a different set of taxa and is renamed E_(1+3)_. Edges that are not part of the donor/recipient pair or ancestral to these edges are not affected by the inferred transfer event.

While many tree comparison metrics exist [15-18], the subtree prune and regraft (SPR) distance [19-21] is most relevant to the inference of LGT events. A *subtree prune and regraft *operation on a binary tree T is performed by cutting any edge and thereby pruning subtree t, and then regrafting the subtree by the same cut edge to a new vertex obtained by dividing a pre-existing edge in T-t (Figure 1). Forced contraction is also applied to maintain the binary property [20]. In the context of an LGT event, the edge that is regrafted onto corresponds to the donor taxon, while the edge that is cut corresponds to the position of the recipient taxon. An *edit path *is any set of such SPR operations that can be applied to a reference tree to yield a topology that is congruent with the inferred protein tree. By extension, an *optimal edit path *is one of the (possibly many) edit paths that is minimal or most-parsimonious for a given reference and test tree, such that there exists no edit path solution requiring fewer edit operations. The length of the optimal edit path is the minimal *SPR distance *or *edit distance *between the two trees.

The computational complexity of calculating the SPR distance between unrooted binary trees is unknown. A proof of NP-hardness was given in Hein et al. [19], but was subsequently noted to be incorrect by Allen and Steel [20], who further showed that the related *tree bisection and reconnection *(TBR) distance problem is NP-hard but fixed parameter tractable (FPT) for unrooted binary trees. Bordewich et al. [21] showed that computing the SPR distance between rooted binary trees is also NP-hard. The problem that we are attempting to solve here is slightly different from the above in that we have a rooted binary reference tree and an unrooted, possibly multificating, test tree. The bipartition measure we use to compare trees treats the reference tree as if it were unrooted and multifurcating when comparing it to the test tree. Thus the problem we are attempting to solve is the SPR distance between unrooted multifurcating trees. The complexity of this problem is open, but given the above results it is very likely to be NP-hard.

A variety of algorithms for calculating or approximating SPR and other distance measures between trees have been proposed in the literature. Hein et al. [19] gave an algorithm that claimed to approximate SPR distance between rooted phylogenetic trees to within a factor of 3. However, Allen and Steel [20] showed that it is actually the tree bisection and reconnection (TBR) distance that is being calculated, and in Rodrigues et al. [22] the approximation factor was shown to be 4. LatTrans [23] calculates the minimum number of SPR operations in the path between two rooted binary phylogenetic trees subject to certain direction of time constraints. They identify two ways in which the (augmented) reference tree and the test tree can disagree (I-fat and H-fat vertices), and give corresponding SPR operations to fix them (I-moves and H-moves). The algorithm is then to find an I-fat or H-fat vertex, fix it, and then recurse, checking that cycles (time violations) are not introduced. The case where there may be multiple copies of a gene in a given species is also considered. The algorithm has been used to identify up to 20 transfers in 300 leaves [24], but in 1% of trials, the algorithm did not find a valid scenario with the real number of transfers.

The HorizStory algorithm [25] approximates the SPR distance between rooted and possibly multifurcating phylogenetic trees. The algorithm works by first eliminating rooted subtrees in the gene and reference trees that are in complete agreement, i.e. are identical. SPR moves are then recursively proposed on the remaining trees until they are brought into agreement. A unique feature of the algorithm is that is can propose *phantom sister *taxa, which are not represented in the reference tree because of incomplete taxon sampling, but donate genes to taxa that are represented in the analysis. While this is a realistic innovation that was not implemented in previous algorithms, a given set of lateral transfers involving no phantom sisters can often be "explained" by a smaller set of transfers involving phantom sisters, in which case the algorithm will not find this larger set.

We have developed a new program EEEP (Efficient Evaluation of Edit Paths) for the inference of edit paths which imposes evolutionarily reasonable constraints on the search space of edits to reduce the overall computational burden while retaining the ability to produce accurate solution sets. An important feature of EEEP is the ability to partition the reference tree into *regions of discordance*, which are reconciled internally with no LGT events permitted between regions. We also introduce the idea of tree distance *ratchets*, where proposed SPR operations on a reference tree are only accepted if the resulting tree is more similar to the test tree (under a *strict *ratchet), or at least as similar (under a *permissive *ratchet). The partitioning of the reference tree and the use of tree distance ratchets do not always yield a complete solution set, but can reduce the running time of the algorithm by several orders of magnitude. The EEEP algorithm has been developed to address some of the problems outlined above, by considering unrooted, possibly multifurcating test trees while imposing time constraints on edit operations that are implied by a rooted reference tree. A precursor to EEEP was used to extract edit paths from a set of 144 prokaryotic genomes [9], with frequently observed edits assigned to 'highways' of LGT between organisms. This precursor version lacked many features that are implemented in the present release, including the permissive ratchets described below, and the ability to remove time constraints on edit operations, which is now a core component of the ratchets. In the analysis presented here, we use random trees to compare the performance of EEEP to that of two other recent LGT detection algorithms, and use EEEP to recover edit paths for the large set of protein trees described in Beiko et al. [9]. We compare the edit distances inferred from this data set with similar distances derived from random trees to assess the extent to which vertical or horizontal signal dominates in the protein trees. Finally, examination of the distribution of phylogenetic distances spanned by inferred LGT events is used to determine whether most or all inferred events occur between closely related taxa (as theory would suggest) or if long-distance transfers are also implied.

## Results and Discussion

### Benchmarking of EEEP

The performance of EEEP was evaluated with both simulated and empirical data. Random rooted and unrooted trees were generated and the performance (time taken and number of edit paths returned) of EEEP was compared to that of LatTrans and HorizStory for strictly bifurcating trees.

**Figure 2:**
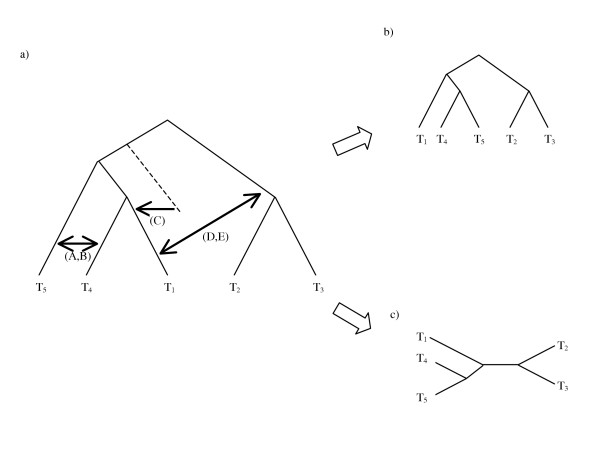
**Phylogenetic tree reconciliations proposed by LatTrans, HorizStory, and EEEP**. Different sets of edit operations, indicated by arrows marked A through E, are proposed to reconcile the reference tree (a) with either a rooted (b) or an unrooted (c) test tree. As described in the main text, LatTrans proposes edits A and B, while HorizStory proposes these two as well as the phantom sister edit C. EEEP with time constraints will propose edits A, B, D, and E, while removing the time constraint allows donation of genetic material from ancestor to descendant, which is analogous to edit C.

The following example, illustrated in Figure 2, shows the differences between the approaches of EEEP, HorizStory and LatTrans, as applied to a reference tree (Figure 2a) and corresponding rooted (Figure 2b) or unrooted (Figure 2c) test trees. The differences between the trees may be resolved by a single lateral transfer in a number of ways, which are indicated with arrows in the figure. LatTrans proposes transfer scenarios A and B: the parent edge of leaf 4 donates to the parent edge of leaf 5, or vice versa. HorizStory proposes three transfer scenarios, the first two (A and B) as for LatTrans, and the third (scenario C) being a phantom sister transfer. The phantom sister edge joins the reference tree between the root and the pendant subtree covering taxa 1, 4, and 5, and this edge donates to the parent edge of leaf 1. The effect is to cut the parent edge of leaf 1 and reattach it above leaves 4 and 5. Without the phantom sister interpretation this would represent a donation to descendant time violation. For EEEP with time constraints, four solutions are found: A and B as in LatTrans and HorizStory, and two further scenarios (D and E) where the edge joining the root of the reference tree to the pendant covering taxa 2 and 3 donates to the parent edge of 1, and vice versa. LatTrans and HorizStory do not propose these solutions, since scenarios D and E lead to a reference tree with a misplaced root. A non-time constrained EEEP search finds a further solution which corresponds to scenario C, the phantom sister solution found by HorizStory.

**Figure 3:**
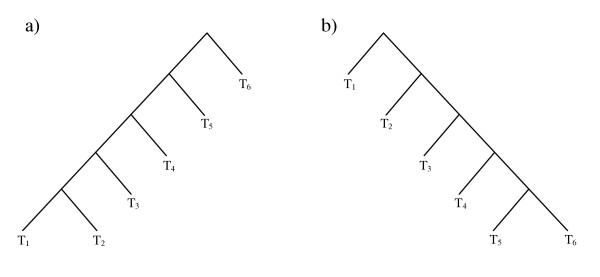
**Effect of a misplaced root on inferred edit paths**. Trees (a) and (b) are identical if the rooting of one or the other is ignored, as would be the case in EEEP, and no edit path reconciliation is necessary. However, LatTrans and HorizStory, which both require a rooted test tree, would be adversely affected by misplacement of the root, since four edits are required to reconcile trees (a) and (b) if the rooting is preserved.

Another difference between the algorithms is the requirement that the test tree be rooted. An example of the effect of root placement on path recovery is shown in Figure 3. The reference (Figure 3a) and test (Figure 3b) trees differ only in the positioning of the root, so EEEP, which does not consider the rooting of the test tree, would not need to produce an edit path at all. Conversely, LatTrans and HorizStory would need to infer four sequential transfers to reconcile the first tree with the second, since taxa T_2 _through T_5 _would all need to be transferred to the other side of the root (the side containing taxon T_6_), one at a time.

Random rooted binary phylogenetic trees were generated for n taxa as follows. A bipartition on the integers from 1 to n was created by randomly cutting the list into two non-empty parts. This bipartition then represents the edges adjacent to the root node of the tree being constructed. Each of the two induced partitions was then randomly split into two lists to create a further bipartitioning of these sets. New bipartitions were then created recursively by cutting elements of previously created bipartitions into two sets until the bipartitions only consisted of singleton elements. Thus the tree was created by starting at the root and creating bipartitions (edges) until the leaf nodes were reached.

Sets of transfers were then applied to the rooted binary phylogenetic trees by choosing random donor and recipient edges to generate test trees. No attempt was made to avoid time violations in generating these random edits. In the case of EEEP the test trees were then unrooted. The leaf and transfer combinations that were tested are shown in Table 1: for each of these combinations, 10 reference and test trees were generated, giving in total 320 reference and test tree pairs tested. The programs were benchmarked on a cluster of 2.0 GHz AMD Opteron computers running Linux. Each process was limited to completing within 5 hours and using at most 3.9 GB of RAM.

**Table 1:** Benchmarking LatTrans, HorizStory and EEEP. Entries show the percentage of cases for which a solution was found, given the constraints of 4 gigabytes of RAM and five hours of running time. Ten reference tree – test tree pairs were generated for each leaf number – transfer number pair, giving 320 tests in all.

**# Leaf (# transfers)**	**LatTrans**	**HorizStory**	**EEEP**
5 (1,2)	100	100	100
10 (1,2,3,4)	100	100	100
15 (1,2,4,6)	100	100	95
20 (1,2,4,6)	100	92.5	90
30 (1,2,4,6)	100	80	92.5
50 (1,2,4,6)	100	60	85
75 (1,2,4,6)	100	50	87.5
100 (1,2,4,6,8,10)	96.7	33.3	70

Comparisons of the performance of EEEP, HorizStory and LatTrans on recovering edit paths for varying numbers of leaves and transfers in randomly generated trees are shown in Table 1. For HorizStory the cases for which a solution was not found were all due to reaching the time limit of 5 hours. The maximum RAM used by HorizStory was 432 MB. For EEEP, the limiting factor was RAM, with no search taking more than 3 hours. LatTrans never exceeded the RAM or time limits, and the small number of cases where no solution was found were likely due to limits in the heuristics of the algorithm. Overall, LatTrans found a solution in 99.4% of cases, HorizStory in 72.8% and EEEP in 88.1%. LatTrans performed remarkably well on every tree size, failing only in a small minority of cases on the set of 100-taxon trees. HorizStory recovered an edit path from every tree of size 5, 10, and 15, but many of the edit paths in larger trees could not be recovered in less than five hours.

**Figure 4:**
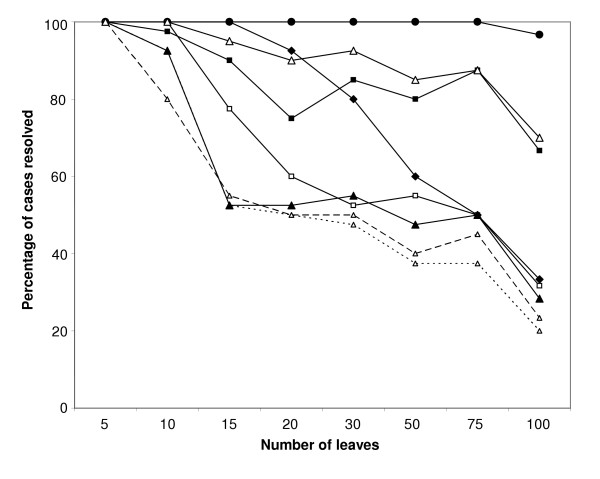
**Successful recovery of edit paths by LatTrans, HorizStory, and EEEP**. The percentage of edit paths recovered from random trees of several different sizes are shown for LatTrans (filled circles) and HorizStory (filled diamonds), and for several different types of EEEP run ('standard' run with time constraints, partitioning and no ratchet – filled triangles; strict test tree ratchet – filled squares; permissive test tree ratchet – open squares; time unconstrained runs – open triangles with long dashed line; unpartitioned runs – open triangles with short dashed line). Open triangles connected by a solid line indicate the percentage of cases where at least one EEEP run was able to recover an edit path. Reference tree ratchets are not shown because the difference in edit path recovery between reference tree and test tree ratchets was never greater than 5%. The runs summarized in this figure were all limited to a maximum of 4 gigabytes of RAM and 5 hours of running time (see manuscript for details).

The success of several different types of EEEP run, as well as HorizStory and LatTrans, in recovering edit paths from trees of various sizes is shown in Figure 4. EEEP runs performed without ratchets failed to recover edit paths from many trees of size 15 or greater, with the worst performance obtained from runs that did not partition trees into regions of discordance (see Methods) and those that did not make use of time constraints. While the performance of EEEP dropped with increasing tree size, the use of ratchets substantially improved the recovery of edit paths, with the permissive test tree ratchet yielding a modest improvement over the standard EEEP run, and the strict ratchet recovering paths from > 80% of all trees containing 75 or fewer taxa, and 66% of all trees with 100 taxa. We assessed the ability of each of the four types of ratchet to recover the correct edit distance as determined by the unratcheted runs. For tree comparisons in which the unratcheted run returned a solution, each type of ratchet was able to recover the correct edit distance from >90% of all data sets, with both permissive ratchets yielding better accuracy (97.1% for both reference and test tree distances) than the strict reference tree (93.8%) and test tree (94.1%) ratchets. Thus, the ratchets provide a considerable increase in speed, with a small sacrifice of accuracy.

### Phylogenomic analysis of 144 prokaryotic taxa

EEEP was also used to infer pathways of LGT by comparing a set of 22 432 protein test trees derived from the microbial data set described in Beiko et al. [9], to a reference supertree constructed using the MRP algorithm [26] and rooted to follow the (by no means universally accepted) paradigm of Bacteria and Archaea as separate, monophyletic domains [27-29]. These trees were inferred from aligned, putatively orthologous sequences using version 3.04 beta of the MrBayes program [30], which samples from a likelihood distribution of trees and generates a posterior probability estimate for each possible bipartitioning of taxa. We considered only those bipartitions with a posterior probability (PP) of 0.95 or greater, which immediately eliminated 2740 data sets from consideration, since the inferred trees for these sets had no bipartitions with support above this threshold. While some of the differences between the protein trees and reference supertree are likely due to violations of phylogenetic assumptions or failure of ortholog mapping and sequence alignment, an extensive set of statistical tests and parsimony analysis of insert/delete states were used in Beiko et al. [9] to show that discordant trees were not simply a consequence of inadequate methods or biased sequenced evolution.

The 19 672 trees with at least one bipartition supported at PP = 0.95 were submitted individually to EEEP, with five different types of ratchet constraints applied in separate runs: a) no constraint; b) a permissive test tree distance ratchet; c) a strict test tree distance ratchet; d) a permissive reference tree distance ratchet; and e) a strict reference tree distance ratchet. These runs were constrained to run within 4 GB of RAM, and the success rate of each method in recovering a solution was assessed. The edit distance and path properties of inferred protein trees were compared with similar properties of randomly generated trees. In the majority of cases, EEEP runs of even the largest data sets completed in less than 30 minutes, either successfully returning a set of edit paths, or reaching the imposed memory limit and terminating.

**Figure 5:**
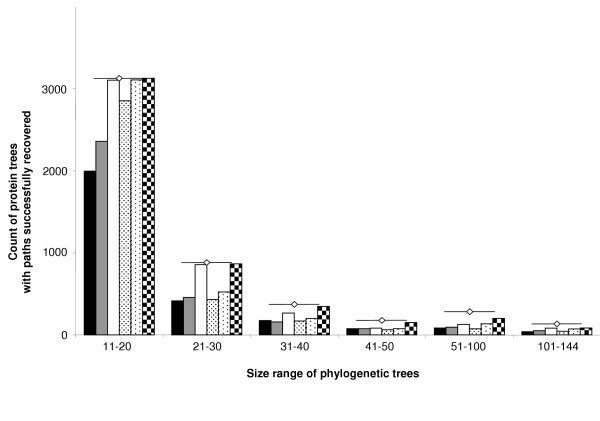
**Recovery of edit paths from inferred protein trees, grouped by size**. Five different EEEP settings were used to recover edit paths from 19 672 inferred protein trees with at least one resolved bipartition, via comparison with the inferred MRP supertree. Five types of bar, ordered from left to right, represent 'standard' EEEP runs with no ratchet, a permissive test tree ratchet, a strict test tree ratchet, a permissive reference tree ratchet, and a strict reference tree ratchet. The final, checkered bar for each category represents the total number of cases where at least one type of EEEP run recovered an edit path solution. The total number of protein trees in each size class (*e.g*., 11–20) is indicated by horizontal lines with a centered open diamond.

Figure 5 shows the success rate of five different types of EEEP run, with varying levels of constraint on the acceptance of edit operations. Only the permissive reference tree ratchet did not recover most-parsimonious edit paths for every protein tree of size 4–10, failing to return a solution for 12 out of 796 ten-taxon protein trees. In every other pooled set, the strict ratchets returned solutions for the largest percentage of protein trees, while the unconstrained runs were least successful in recovering edit paths. There is a tendency toward the recovery of fewer edit solutions from larger data sets, but the recovery rate from protein trees of size 101–144 (33%–65%) was actually better than for protein trees of size 51–100 (28%–51%). An edit path was recovered by at least one method in 19 544 of 19 672 cases. As mentioned above, memory consumption, rather than computational time, was the reason for non-recovery of solutions from the other 128 protein trees.

**Figure 6:**
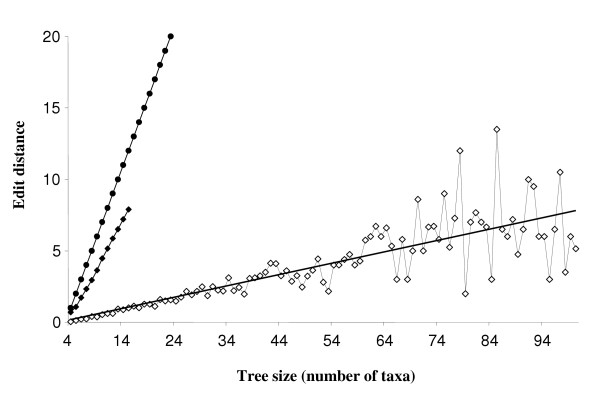
**Theoretical maximum, and observed mean edit path length for random and inferred protein trees of different sizes**. Filled circles show the maximum possible most-parsimonious edit path length for trees with *n *taxa (= *n *- 3). Filled diamonds indicate the mean edit distance recovered from comparisons between random pairs of trees with up to 15 taxa, with each tree size replicated 500 times. Open diamonds show the mean edit distance recovered for protein trees of size 4 to 100, with the linear best-fit relationship for the points in this range shown (y = 0.080x + 0.108, R^2 ^= 0.656).

The number of inferred LGT events within a given set of trees expresses the extent to which horizontal acquisition of genes has influenced genome evolution in the represented taxa. Simulations of random LGT events via multiple SPR permutations can be used to distinguish empirical trees from random trees, since the former should only approximate the latter when LGT is rampant and sufficiently random [31]. Thus, if random LGT events dominate a given dataset, then the SPR distance between any inferred tree and the reference tree should be similar to the average distance between pairs of random trees. If vertical inheritance (or some other cohesive signal) dominates in a set of inferred trees, then the number of edit paths observed should be smaller. To assess the significance of the average similarity between reference and test trees, we should know the distribution of edit distances between pairs of random trees. The average distance between a pair of trees is not known, though Song [32] presents a formula for the number of trees that are separated from a given tree by a single SPR operation. We must therefore resort to building an empirical distribution from random pairs of trees. Another problem is the bias toward non-recovery of edit paths from larger trees will yield underestimates of mean edit distance for these sets. Consequently, we performed comparisons between many pairs of random trees to generate a background distribution of edit distances between trees, and concentrated on trees with 15 or fewer taxa, in a range where EEEP was able to recover edit path solutions for every protein tree tested above. Figure 6 shows the mean edit path length recovered for each size class of protein trees, compared to the maximum possible edit path length (= *n *- 3) and the mean edit path length recovered from comparisons between 500 pairs of random trees. For comparisons between random trees, each unit increase in taxon size yields an increase of 0.675 in average edit distance (R^2 ^= 0.998). When protein trees of size 4 to 15 are considered, each unit increase yields an increase of only 0.087 in average edit distance (R^2 ^= 0.968). Extending the model to include protein trees of sizes 16 to 100 yields a similar slope (0.080), with a reduced linear fit coefficient of 0.656 due to instability in mean estimates for larger protein tree sizes. Since the slope of this relationship between inferred protein trees is approximately 1/8 of the equivalent relationship between random trees, it is clear that there is a strong cohesive, likely vertical, signal in the protein trees.

**Figure 7:**
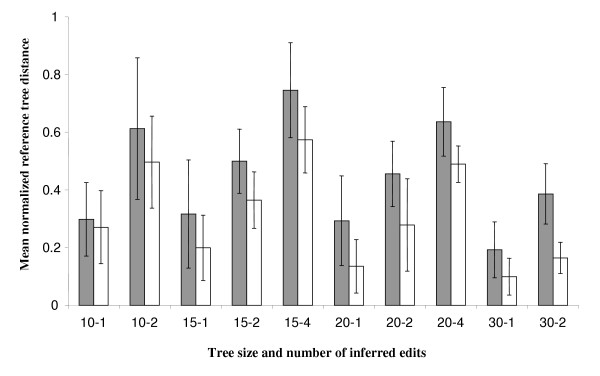
**Mean normalized reference tree distance for inferred protein trees and random trees with different numbers of taxa and edits**. Each pair of vertical bars indicates the mean ± standard deviation of the mean normalized reference tree distance (defined in the text) for 10 pairs of random trees (gray bars) and for >5 protein tree/MRP supertree pairs (white bars). Pairs of numbers on the x-axis indicate combinations of tree size – number of inferred edits.

Tree edits induced by an SPR operation can implicate lineages that are separated by any number of internal branches. This concept of 'short' versus 'long' edits is captured in measures such as the nearest-neighbor interchange (NNI) distance [33-35], but must be assessed indirectly in the case of SPR operations. The count or total length of internal branches separating the donor and recipient lineages can be used to assess the 'length' of an edit operation [36]. For a fixed number of edits, the number of regions of discordance and other tree distance measures can be used as well: long-distance edits will tend to overlap, yielding fewer and larger regions of discordance, and bipartition-based measures such as the Robinson-Foulds distance will be larger due to the greater number of bipartitions disrupted by edit operations. Figure 7 shows the normalized reference tree distance (number of discordant reference tree bipartitions divided by the count of discordant and concordant reference tree bipartitions) for several combinations of tree size and number of edits, for inferred protein trees and the randomly permuted trees described earlier in "Benchmarking of EEEP" above. While the average normalized reference tree distance is invariably larger in the set of randomly permuted trees, there is a wide distribution of normalized distances in both types of tree, evidenced by the large standard deviations associated with each group. If α = 0.05 is used as the cutoff for statistical significance, and divided by ten to yield a Bonneferroni-corrected threshold of 0.005, then in only three out of ten classes (15 taxa / 2 edits, 20 taxa / 2 edits, and 30 taxa / 2 edits) is the difference between experimental and random trees statistically significant. Thus, edits inferred from protein trees tend to be 'shorter' on average than randomly induced edits, but there is considerable overlap between the distributions of the two sets. There is a similar tendency of random edits to associate into fewer regions of discordance. When trees with > 1 edit (and therefore potentially > 1 region of discordance) were considered, in all cases a majority of trees contained only a single discordant region. However, a larger minority of protein trees contained multiple regions of discordance (typically 30–50%) than did the randomly permuted trees (typically 10–20%). This observation is consistent with the ability of EEEP to recover edit path solutions for very large protein trees with > 10 edits, due to partitioning of the tree into multiple regions of discordance, while randomly permuted trees of a similar size often could not be resolved.

While inferred protein trees showed a high degree of similarity (characterized by low edit distance) to a reference supertree, the number of internal edges spanned by inferred edit operations was only marginally shorter than those generated under a random model. While statistical and biological hypotheses tend to predict 'local' topological rearrangements (characterized by low NNI distance), in our data set we frequently inferred LGT events between prokaryotic phyla and between the bacterial and archaeal domains. The dissimilarity between orthologous sequences from different phyla precludes homologous recombination, which requires a high degree of sequence similarity, so these transfers require illegitimate recombination to integrate the donor DNA into the recipient genome [37-40]. The existence of these transfers is supported by phylogenetic profile analysis, which shows a patchy distribution for many sets of genes that cannot be explained through loss alone.

### Relationship between discordant bipartitions and edit distance

**Figure 8:**
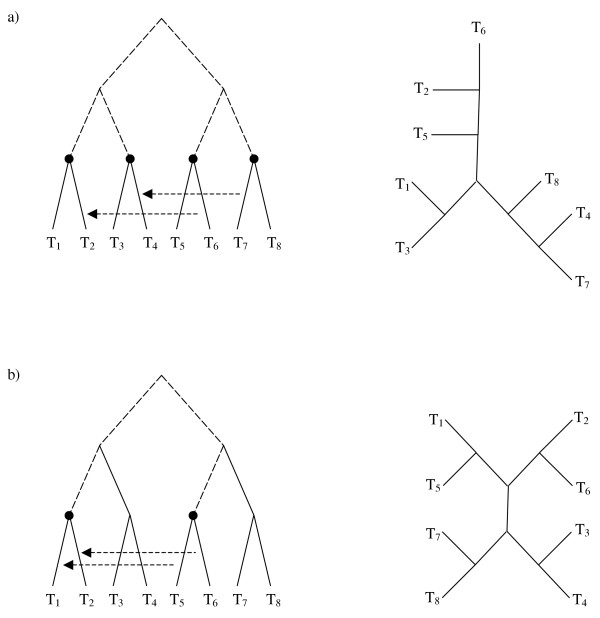
**Trails of destruction for two pairs of six-taxon trees**. Two pairs of reference and test trees are shown: in both cases, the test tree is the consequence of two edit operations on the corresponding reference tree. The edits are indicated with boldface arrows, with the arrowhead pointing to the recipient lineage. The trails of destruction are composed of the edges in each reference or test tree which imply bipartitionings of taxa that are incompatible with the other tree. These incompatible edges are drawn with dashed lines in the figure, and the endpoints indicated with filled circles. In Figure 8a, there are four endpoints on the trail of destruction, thus identifying the minimum number of edit operations as 2. The trail in Figure 8b has only two endpoints, so in this case the minimal edit distance of 1 suggested by the endpoints is not the correct number of edits.

When an SPR operation is performed on a reference tree, the internal edges in the original tree that connect the donor and recipient taxa will be incompatible with edges in the derived tree, since the bipartitions of taxa implied by these edges are not found in the new tree. This chain of internal edges defines a 'trail of destruction' that can be used to set minimum bounds on the number of edit operations needed to reconcile the two trees (described in detail in Methods). For each protein tree that could be reconciled with the reference tree by EEEP, we compared the minimum edit distance recovered by EEEP with the total number of endpoints associated with the trails of destruction in each protein tree. For the 3714 protein trees with an edit distance of 1 relative to the supertree, the result is trivial: a single edit operation on a reference tree induces a trail of destruction containing exactly two endpoints, with the donor and recipient lineages at opposite ends of the chain. Each of these trees must therefore contain a single linear trail. Each subsequent edit operation can increase the number of endpoints by 0, 1, or 2 with respect to the original reference tree, so among protein trees with an edit distance *D *≥ 2, the number of endpoints is bounded by 2 and 2*D*. If 2*D *or 2*D *- 1 endpoints are observed in comparing a reference with a test tree, then *D *is guaranteed to be the minimal edit distance. Of the 2062 protein trees with D ≥ 2, 873 (42.3%) could be confirmed as minimal edit paths with this approach. Two simple examples of trails of destruction from overlapping edit operations are shown in Figure 8: an example where the number of endpoints is equal to 2*D *(Figure 8a), and a case where the endpoint count is equal only to *D *(Figure 8b).

## Conclusion

All three algorithms tested here on random trees were limited to some extent by computational resources: the amount of RAM was typically limiting for EEEP, and running time for HorizStory. LatTrans is extremely fast and efficient relative to the other algorithms, but its requirement of strictly bifurcating, rooted trees makes it an inappropriate choice for phylogenomic analyses. The most straightforward approach to increasing the range of problems that can be resolved with EEEP is through the use of faster computers with more memory, and parallelization. However, the 'trails of destruction' give interesting clues about the nature and number of SPR operations that are required, and should be considered as a possible source of new ways to refine the search for most-parsimonious edit paths.

We have used EEEP to reconcile a reference supertree comprising 144 organisms with 19 672 microbial protein trees as part of a large phylogenetic study [9]. Not surprisingly, for these data the inferred protein trees were far more similar to each other and to the reference supertree than were randomly generated pairs of reference and test trees. This similarity reflects cohesive signal in the inferred protein trees that is congruent with the organismal history, e.g. 'vertical' inheritance from parent to offspring. Since vertical signal dominates in this data set, there is no evidence that LGT has destroyed the organismal signature that should be evident in sequence phylogenies. As statistical tests and corrections for violations of basic phylogenetic assumptions improve [28,41-44], and as hypotheses about the 'transferability' of different types of gene are refined [2,7,9,45], the underlying organismal history initially suggested by 16S rRNA analysis and refined using other data sets should solidify.

### Final words – interpreting edit paths

A final challenge in the inference of edit paths is the interpretation of multiple, equally parsimonious edit paths. The most frequent examples of multiple edit paths involve simple permutations of the same set of edits, which imply that there is no time constraint on the order in which those edits can occur. Also common are symmetrical exchanges, where a gene transfer event involving two taxa yields the same effect on tree topology regardless of which taxon is the donor and which the recipient. In these cases, information about the *partners *obligately involved in the exchange may be clear, and interpretable in light of hypotheses about preferential gene sharing between organisms, but the *direction *of transfer cannot be deduced solely from the tree topology. Finally, a given comparison of trees can produce multiple edit paths that suggest partially overlapping or non-overlapping sets of transfer partners. These cases typically identify a small set of possible exchange partners, but fail to uniquely identify each pair of taxa participating in a transfer. Analysis of large sets of obligate and possible transfers can reveal trends that correspond to 'highways' of gene sharing [9], but conclusions about the sharing history of individual genes may be unclear.

Can other information be used to uniquely identify the donor and recipient implicated in an LGT event? While EEEP, HorizStory and LatTrans consider only the branching order of phylogenetic trees, other features of the trees and the underlying sequences can potentially convey useful information as well. If genome-to-genome 'distances' are implied either in the reference tree or through some other method, then sequence-to-sequence distances that are much shorter than expected may identify the partners in a recent genetic transfer event. This type of assessment relies on the assumption that sequences have evolved in a clock-like fashion, which may be frequently violated if transferred sequences evolve more rapidly in the recipient genome due to amelioration pressures or functional duplication [46]. The topological analysis performed by EEEP may also be complemented by the use of surrogate methods which consider the deviation of residue composition within a gene from that of the 'background' genome sequence [45-47]. Most of these methods can be applied to a single genome, so the detection of recently acquired sequences is not dependent on having a relative of the donor sampled as well. However, since these methods typically rely on genomic sequence bias to generate a null distribution, they are particularly sensitive to amelioration [46] and the overlap among the predictions of different methods is quite poor [48]. Given the evidence that different surrogate methods may detect different types of LGT events, or events of different ages [49], the best strategy is to carefully consider the results of several different approaches.

## Methods

### Tree features

We require a method to compare a rooted binary reference tree and an unrooted multifurcating test tree to test when they are topologically congruent. The approach taken by EEEP is to consider the *bipartitions *induced by the edges of reference and test trees. The key to topological comparisons is the subdivision of reference tree bipartitions into those that are concordant and discordant with respect to the test tree. A reference tree bipartition splits the set of taxa T into two sets A = {A_1_, A_2_, ..., A_m_}, and B = {B_1_, B_2_, ..., B_n_}. This bipartition is *concordant *if a bipartition exists within the test tree which implies the exact same split of taxa, with the exception of those taxa that are not present in the test tree. The reference bipartition is *discordant *if both sets of taxa implied by one or more test tree bipartitions contain representatives of set A and set B. A reference tree bipartition may be neither concordant nor discordant if the test tree contains multifurcating nodes, and none of the supported test tree bipartitions satisfy the criteria for concordance or discordance. The goal of EEEP is to reconcile a reference tree with a test tree by performing SPR operations on the reference tree until no discordant bipartitions remain, and it is completely consistent with the test tree.

An LGT event is the donation of genetic material from a donor lineage in the tree to a recipient lineage, and is represented in our diagrams with an arrow connecting the two lineages that participate in the transfer operation. The consequent SPR operation merges the recipient and donor lineages, which subdivides the donor edge (E_4 _in Figure 1) into a descendent edge which implies the same bipartitioning as the donor, and an ancestral edge that is the parent of the new merged donor-recipient clade (E_(3+4) _in Figure 1). The parent edge of the old recipient (E_0 _in Figure 1) is lost. Every other bipartition corresponding to an edge that is ancestral exclusively to either the donor or the recipient is modified, as the recipient clade is exchanged between the two bipartitions implied by these edges. The bipartitions induced by the donor and recipient edges, and any of their descendants, are not affected by the SPR event, nor are any edges ancestral to the common ancestor of the donor and the recipient edges. A special case of the SPR event occurs when the donor and recipient taxa are already sisters. While LGT is likely to be most common between closely related genomes due to the propensity of similar DNA sequences toward homologous recombination, such events have no impact on the resulting phylogenetic tree and as such cannot be detected using topological comparisons. Since they are unproductive, SPR operations between sister taxa are prohibited by EEEP.

A consequence of comparing many test trees to a single organismal reference is that many or all of the test trees may contain only a subset of the taxa that are covered by the reference tree. In such cases, the reference tree must be projected by removing taxa that are not represented in the test tree, and consolidating the edges that confer the same information as a result. For instance, if a test tree containing only taxa T_1 _- T_6 _was compared to the reference tree shown in Figure 1a, then removal of T_7 _from the reference tree would eliminate edge E_11_, and edges E_5 _and E_10 _would describe the same bipartitioning of taxa. In such a case, E_5 _and E_10 _would be collapsed into an equivalence class, since operations involving one of these edges would yield the exact same tree topology as operations involving the other.

Another important feature of trees induced by one or more LGT events is the set of discordant bipartitions. Suppose we have a reference tree and a test tree on the same set of leaves for which there have been no lateral transfer events. In this case the reference tree and the test tree will be identical. Now suppose there is a reference tree as in Figure 1 and that a single gene transfer event has occurred between two edges. The edges in the original reference tree that are inconsistent with the resulting gene tree are exactly those on the unique path between the edges involved in the transfer. Similarly, the discordant edges in the test tree are those on the path between the donor and recipient points. Hence an LGT event leaves a 'trail of destruction' of discordant edges in the reference and test trees.

If we were given any reference tree and a test tree for which the discordant edges in the reference tree formed a single chain it would be evident that at least one transfer event would be required to reconcile the differences between the trees, and if it were indeed a single transfer event then it must have occurred from and to the ends of the chain. More generally the sub-graph on discordant edges of the reference tree can yield information on the minimum number of transfers required to reconcile the reference and test trees. If the discordant edge subgraph forms a single component, then it follows immediately that the number of transfers required is at least the number of degree 1 nodes (leaves) of the discordant subgraph divided by 2. This is illustrated in Figure 8, where the discordant edges are drawn with dashed lines. There are 4 degree 1 nodes in Figure 8a and 2 such nodes in Figure 8b, hence the minimum number of LGT events is 4/2 = 2 in the first case and 2/2 = 1 in the second. Most generally, if the discordant edge subgraph has multiple components, under the assumption that transfer events have not occurred *between *components, then the minimum number of transfer events is again at least the number of degree 1 nodes of the discordant subgraph divided by 2. The EEEP distribution contains software that will draw a reference/test tree pair, and indicate which edges in each are discordant, to highlight these 'trails of destruction'.

### Constraints on SPR operations

The length of all possible most-parsimonious edit paths between any pair of strictly bifurcating trees covering *n *taxa is between zero and (*n *- 3) [20]. While the scaling of the longest possible most-parsimonious edit path is linear with the number of taxa, the number of possible most-parsimonious edit paths is proportional to the number of possible trees. A tree with *n *taxa contains *n *terminal edges and (*n *- 3) internal edges, with an additional edge induced if the tree is rooted: for instance, the tree in Figure 1a has a total edge count ε of (7 + 4 + 1) = 12. If no constraints are placed on the choice of donor and recipient edges, then a total of ε(ε -1) distinct SPR operations can be carried out on any given tree, reflecting the total number of donor/recipient pairs than can be chosen. In the case of the seven-taxon tree in Figure 1a, a total of (12 × 11)^4 ^= 303595776 edit paths of length 4 can be generated. Constraints on the set of proposed SPR operations must be used to resolve all but the most trivial problems in reasonable time. In EEEP, the edit path determination is carried out in a breadth-first fashion, where candidate SPR operations are performed on the current modified version(s) of the reference tree with an edit path of length *l *before any paths of length *l *+ 1 are considered. In the absence of any knowledge of the total length of the most-parsimonious edit path(s), the breadth-first search is necessary to avoid performing more SPR operations than are necessary in any given edit path.

Two restrictions have a modest impact on the number of cases that must be considered. The first, mentioned above, is the prohibition of transfers between edges that are sisters in the original or modified reference tree, since such transfers yield no change in the tree topology when branch lengths are not considered. Secondly, in an edit path of length > 1, the same edge cannot act as recipient in multiple SPR operations, since the effect of the first such SPR event would be most parsimoniously explained by a single transfer event.

The number of tree and bipartition comparisons that needs to be considered can be considerably reduced by identifying and eliminating regions of the reference tree that will definitely not occur in the most-parsimonious edit path, and by partitioning the tree into regions of discordance which will not be bridged by SPR operations. After zero or more edits, each derived reference tree topology is compared to the test tree to determine whether any concordant pendant edges are present. Allen and Steel [20] showed that for unrooted binary trees, any pendant subtrees that are completely concordant can be excluded from further SPR operations in searching for the SPR distance (though not necessarily the complete set of paths), and the same result holds for unrooted multifurcating trees. If such edges are observed, the entire set of bipartitions and taxa in the subtree are replaced with a single taxon representing the entire group, which reduces the number of comparisons that are necessary. In some cases, the reference tree can also be cut into two or more 'regions' if the discordant edges are interspersed with concordant ones. A given region will have either a concordant edge or the root of the tree as its last common ancestor, and concordant edges (which may be terminal and subtend only single taxa) as its descendants. By prohibiting SPR exchanges where the donor and recipient are from different regions of the tree, the number of edit operations that needs to be considered can be reduced dramatically. However, partitioning the tree in this manner yields a final edit path solution that may not be complete: some alternatives among the most-parsimonious set of edit paths for unusual trees may in fact implicate donor and recipient lineages from different regions of discordance.

The time constraints implied by a rooted tree can be used to constrain the set of legal donor/recipient pairs. If a given edge has been either the donor or recipient in an SPR operation (and thus implicitly participated in an LGT event), the ancestors of that edge are prevented from participating in subsequent SPR operations, since this order of events would imply that a transfer event between a pair of ancestors occurred *after *an exchange between their descendants. Similarly, donor/recipient pairs where one edge is an ancestor of the other are prohibited, since transfers must occur between contemporary genomes. However, this last restriction can be relaxed in EEEP, because an SPR event where the donor is an ancestor of the recipient could imply a contemporary donor or "phantom sister" [25] that is not represented in the reference or test trees, due either to limited sampling or extinction. Unlike the donor taxon, the recipient or one of its descendants *must *be present in the tree for the transfer event to be detected.

**Figure 9:**
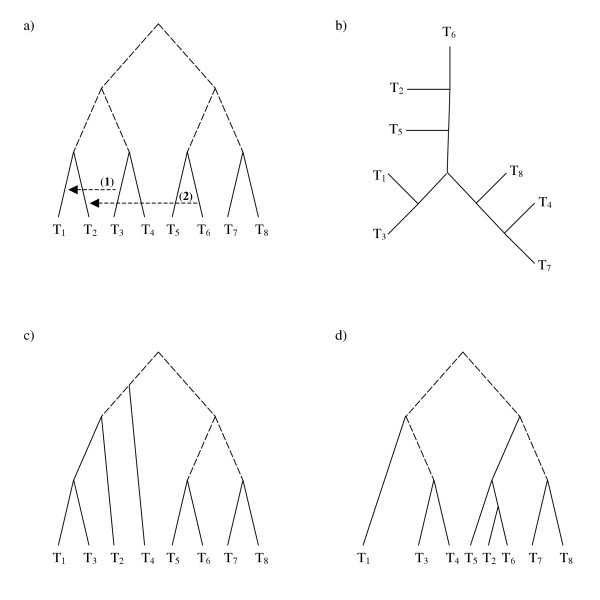
**Ratchets based on reference tree distance**. Comparison of a rooted reference tree (a) with an unrooted test tree (b) shows that five internal edges in the reference tree imply bipartitionings of taxa that are not consistent with the test tree (note that since they imply the exact same bipartition, the two edges connected to the root of the reference tree only contribute a single count), corresponding to a reference tree distance of 5. Two SPR operations that could be proposed by EEEP are indicated with dashed arrows: the resulting modified reference trees are indicated for arrow number 1 in panel (c), and for arrow number 2 in panel (d). These two trees have reference tree distances of 4 and 3 respectively from the test tree, and are therefore not treated equally if a ratchet is being used. Under a permissive ratchet, both edits would be accepted and used for subsequent SPR moves, because both yielded a decrease in the overall reference tree distance. However, under a strict ratchet, only the edit that yielded a reference tree distance of 3 would be accepted, because it yielded the largest decrease in reference tree distance.

There are many ways to express the degree of difference between a pair of trees. Any of these measures (save the edit distance itself) can be used to assess whether a modified reference tree obtained after an edit operation is more or less similar to the test tree than the pre-modification tree. Two types of ratchet can be imposed: a weaker or 'permissive' constraint that prohibits edits which fail to yield increased similarity between the reference and test trees, and a 'strict' constraint that permits only the best (i.e., most-similar) trees from the current round to be considered in the next round of operations. None of these ratchets has yet been proven to yield an optimal and complete set of solutions (and many clearly do not), but in practice they usually yield a substantial increase in computational efficiency and a solution set that is identical to a ratchet-free run. Also, as mentioned previously, by counting terminal nodes in the discordant edge subgraphs, in many circumstances we can be sure of having found a shortest edit path. Two types of ratchet are currently implemented in EEEP: the *test tree bipartition distance *counts the number of bipartitions from the test tree that are discordant with the current reference tree, and the *reference tree bipartition distance *counts the number of bipartitions from the reference tree that are discordant with the current test tree. An example of the reference tree distance ratchet is shown in Figure 9. If a ratchet is used, then the time constraints above must be temporarily discarded, since the SPR operations that yield the best improvement in tree similarity may not necessarily occur earlier in the tree (closer to the root) than later operations. Under a time constraint and a ratchet, performing the best move first could preclude subsequent moves closer to the root that yield lesser (but still necessary) improvements. Consequently edit path determination is carried out without the time constraint, but any edit paths obtained at the end are permuted to determine whether a solution does exist that is legal in time.

## Authors' contributions

Both authors contributed to the development, benchmarking, and application of this algorithm to empirical data, and to the writing of the manuscript.

## Availability and requirements

EEEP is released under the GNU General Public License. It has been tested on 32-bit Windows, RedHat Linux and AIX operating systems. Source code and compiled binaries are available from .
